# Lectin Staining Shows no Evidence of Involvement of Glycocalyx/Mucous Layer Carbohydrate Structures in Development of Celiac Disease

**DOI:** 10.3390/nu5114540

**Published:** 2013-11-18

**Authors:** Henrik Toft-Hansen, Christian Nielsen, Matteo Biagini, Steffen Husby, Søren T. Lillevang

**Affiliations:** 1 Hans Christian Andersen Children’s Hospital, Odense University Hospital, Sdr. Boulevard 29, Odense 5000, Denmark; E-Mail: steffen.husby@rsyd.dk; 2 Department of Clinical Immunology, Odense University Hospital, Sdr. Boulevard 29, Odense 5000, Denmark; E-Mails: christian.nielsen@rsyd.dk (C.N.); soren.lillevang@rsyd.dk (S.T.L.); 3 Department of Pathology, Odense University Hospital, J.B. Winsløws Vej 15, 2., Odense 5000, Denmark; E-Mail: matteo.biagini@rsyd.dk

**Keywords:** pediatric celiac disease, type 1 diabetes, duodenal biopsies, glycosylation, lectin staining

## Abstract

The presence of unique carbohydrate structures in the glycocalyx/mucous layer of the intestine may be involved in a susceptibility to celiac disease (CD) by serving as attachment sites for bacteria. This host-microbiota interaction may influence the development of CD and possibly other diseases with autoimmune components. We examined duodenal biopsies from a total of 30 children, of which 10 had both celiac disease (CD) and type 1 diabetes (T1D); 10 had CD alone; and 10 were suspected of having gastrointestinal disease, but had normal duodenal histology (non-CD controls). Patients with both CD and T1D were examined before and after remission following a gluten-free diet. We performed lectin histochemistry using peanut agglutinin (PNA) and Ulex europaeus agglutinin (UEA) staining for Gal-β(1,3)-GalNAc and Fucα1-2Gal-R, respectively, of the glycocalyx/mucous layer. The staining was scored based on dissemination of stained structures on a scale from 0 to 3. Evaluation of the scores revealed no difference between biopsies obtained before and after remission in the group of children with both CD and T1D. A comparison of this pre-remission group with the children who had CD alone or the non-CD controls also showed no significant differences. Based on our material, we found no indication that the presence of Gal-β(1,3)-GalNAc or Fucα1-2Gal-R is involved in the susceptibility to CD, or that the disease process affects the expression of these carbohydrates.

## 1. Introduction

The role of the adaptive immune system in celiac disease (CD) has been studied in detail over the past few decades, and its association with HLA-DQ2 and DQ8 is well established [1]. Association with other genomic regions has been demonstrated, but the individual impact of each region is small, and, in combination, these regions can only explain about 5% of the total genetic disease susceptibility [2]. Recently, there has been a surge of interest in the possible role of the microbiota in the etiology of CD and a wide range of other diseases, particularly with autoimmune components [3,4].

The glycocalyx/mucous layer covers the gastrointestinal tract, and acts as a semi-permeable barrier between the lumen and the epithelium. Bacteria in the host microbiota may use carbohydrate structures in the glycocalyx/mucous layer as attachment sites [5]. Inherited differences in expression of certain carbohydrates could lead to a predisposition to colonization with particular strains of bacteria, which may influence the susceptibility to CD. Alternatively, the presence of certain bacteria independent of inherited factors could cause an environmental predisposition to CD by altering the expression of carbohydrates. Also, the disease process involving inflammation of the gut mucosa could affect the expression of carbohydrates [6].

Bacteria that are decreased or enriched in the gut of CD patients compared to healthy controls could be important in maintaining gut homeostasis in the healthy individuals or in compromising the epithelial function in CD [7]. It has been shown that rod-shaped bacteria were frequently found in the mucosa of pediatric CD patients with active disease, as well as in another group of pediatric CD patients in remission on a gluten-free diet (GFD), though not in controls with no known food intolerances [8]. Since the rod-shaped bacteria were also present in the children in remission, this indicates that CD patients could be genetically predisposed to colonization with these particular bacteria, perhaps due to the expression of particular carbohydrates.

The prevalence of CD in patients with Type 1 Diabetes (T1D) is estimated to be around 8% compared to around 1% in the general population [9]. CD and T1D share genetic risk factors with a strong association to HLA-DQ2 or DQ8 for both diseases [10]. According to Danish guidelines, all children diagnosed with T1D are screened by serology for the presence of CD-associated antibodies. In contrast to children with CD alone, biopsies are also taken after the implementation of a gluten-free diet in children with both diseases. By studying biopsies obtained from the same children with CD and T1D before and after remission, as well as children with CD alone or no CD at all, we aimed to investigate whether or not the expression of unique carbohydrate structures appeared to be genetically determined or affected by the inflammatory status of the tissue.

## 2. Materials and Methods

### 2.1. Study Design

We obtained duodenal biopsies from a total of 30 children. Among this group, 10 had both celiac disease (CD) and type 1 diabetes (T1D) (aged 5–11 years, mean = 8.6); 10 had CD but not T1D (aged 1–14 years, mean = 5.9); and 10 were non-CD controls, verified to have normal duodenal histology (aged 2–17, mean = 7.9), but who could have other gastrointestinal diseases, chiefly gastroesophageal reflux disease. The group with both CD and T1D were examined both before and after remission (2–4 years after first biopsy) following introduction of gluten-free diet (GFD). Biopsies were scored according to the Modified Marsh classification [11] (Table 1).

**Table 1:** Biopsy Marsh scores for individual patients. Group A consists of patients with both celiac disease (CD) and type 1 diabetes, so each patient has two scores: untreated and after remission (GFD). Group B patients have CD only, and Group C patients do not have CD (normal biopsies), but may have other diseases.

Group A Patient ID	CD + T1D Untreated	CD + T1D GFD	Group B Patient ID	CD	Group C Patient ID	Non-CD
A1	3A	0	B1	2	C1	0
A2	3C	0	B2	3B	C2	0
A3	2	0	B3	3C	C3	0
A4	2	0	B4	3B	C4	0
A5	3B	0	B5	3B	C5	0
A6	3C	0	B6	3C	C6	0
A7	3C	0	B7	3C	C7	0
A8	3A	0	B8	3C	C8	0
A9	3B	0	B9	3C	C9	0
A10	3C	0	B10	3C	C10	0

### 2.2. Lectin Histochemistry

We performed lectin histochemistry using peanut agglutinin (PNA) and Ulex europaeus agglutinin (UEA) staining carbohydrates in glycolipids or glycoproteins of the glycocalyx/mucous layer. PNA specifically binds the galactose sequence Gal-β(1,3)-GalNAc, and UEA specifically binds the fucose sequence Fucα1-2Gal-R [12]. Briefly, duodenal biopsies were embedded and snap-frozen in liquid nitrogen immediately after gastroscopy. Cryosections were fixed, followed by blocking of endogen biotin with an Avidin Biotin kit (DAKO, Glostrup, Denmark) and then incubated with biotinylated lectin, rinsed, and followed by incubation with horse radish peroxidase (HRP)-conjugated streptavidin (DAKO). Bound lectins were visualized using the HRP substrate carbazol.

The staining was scored based on dissemination of stained structures in the epithelial cell layer on a scale from 0 to 3, where 0 represents no staining of the epithelial cell layer, 1 represents staining in the cytoplasm of epithelial cells, 2 represents staining of cytoplasm and glycocalyx, and 3 represents staining of cytoplasm and glycocalyx, in addition to villus goblet cells. The observed staining pattern was hierarchical as described. Staining of crypt goblet cells was not considered in the analysis. Intensity of staining was not included in the score.

### 2.3. Ethics

The study was approved by the Regional Committee for Biomedial Research (VF-20050134) and by the Danish Data Protection Agency (DOK 2709660991).

### 2.4. Statistical Methods

Statistical analysis was performed with non-parametrical tests using the GraphPad Prism software [13]. A statistical significance limit of *p* *<* 0.05 was chosen.

## 3. Results

**Figure 1:**
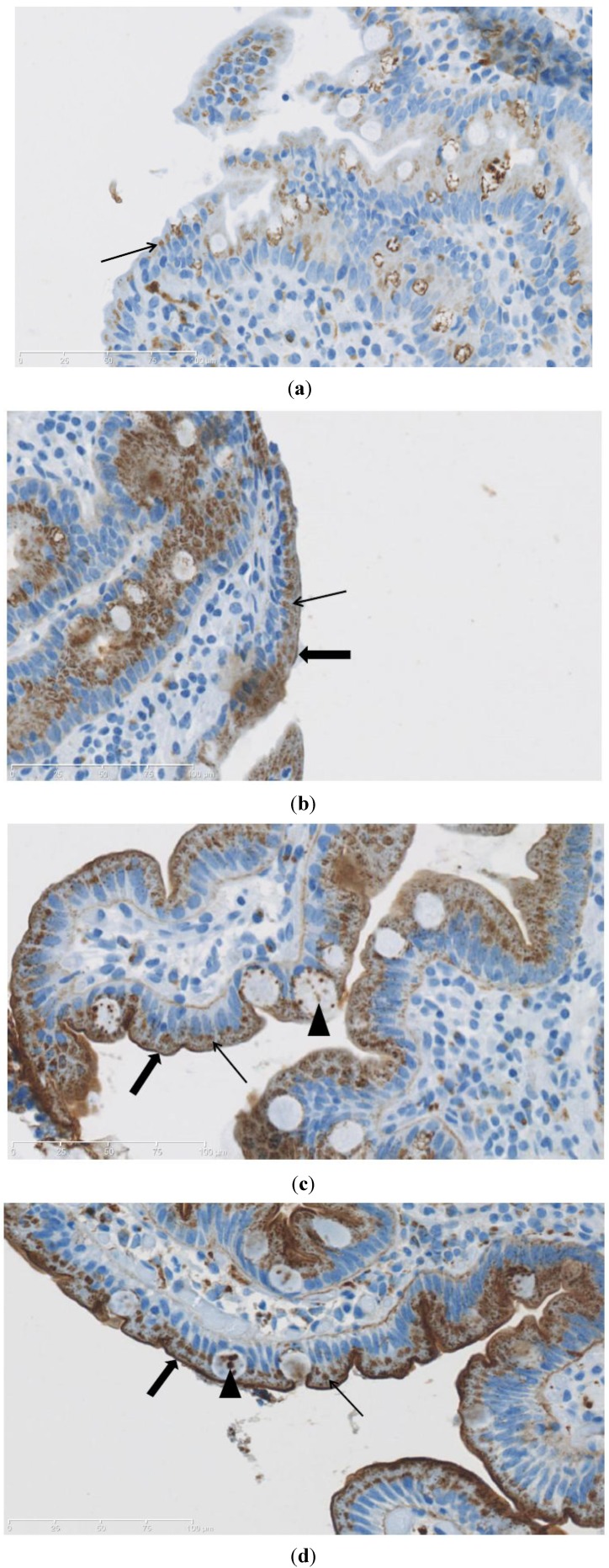
Peanut agglutinin (PNA) staining. (**a**) Patient B3 (CD, untreated), score 1; (**b**) Patient A7 (CD+T1D, untreated), score 2; (**c**) Patient A7 (CD + T1D, on GFD), score 3; (**d**) Patient C2 (non-CD), score 3. Thin arrows mark staining of cytoplasm of epithelial cells. Thick arrows mark staining of glycocalyx. Arrowheads mark staining in goblet cells.

PNA staining patterns are illustrated in Figure 1 with examples of staining in samples from a CD patient (Figure 1a), a CD + T1D patient untreated and treated with a GFD (Figure 1b,c, respectively), and finally a non-CD patient (Figure 1d). Some degree of PNA staining was evident in all samples in all groups (Table 2) with a score of 1 as the lowest (Figure 1a), where only cytoplasmic was observed. Figure 1b,c show PNA staining of biopsies obtained from the same patient (A7) with both CD and T1D before and after treatment with GFD. In the case of patient A7, the score went up from 2 in the untreated state to 3 after treatment, but this was not a consistent trend for this group of patients as a whole. A score of 2 (Figure 1b) includes staining of the brush border as well as cytoplasm, and a score of 3 (Figure 1c,d) indicates additional staining in some, but not necessarily all villus goblet cells. The PNA staining in goblet cells appeared localized to spots inside the cells.

**Table 2:** Peanut agglutinin staining scores. Stained sections of biopsies from patients in Groups A (before and after remission), B, and C were scored as follows: 0 represents no staining of the epithelial cell layer, 1 represents staining in the cytoplasm of epithelial cells, 2 represents staining of cytoplasm and glycocalyx, and 3 represents staining of cytoplasm and glycocalyx, as well as goblet cells.

	Group	A CD + T1D Untreated	A CD + T1D GFD	B CD	C Non-CD
Score					
3		0	2	0	2
2		5	4	1	0
1		5	4	9	8
0		0	0	0	0
Median score		1.5	2	1	1

**Figure 2:**
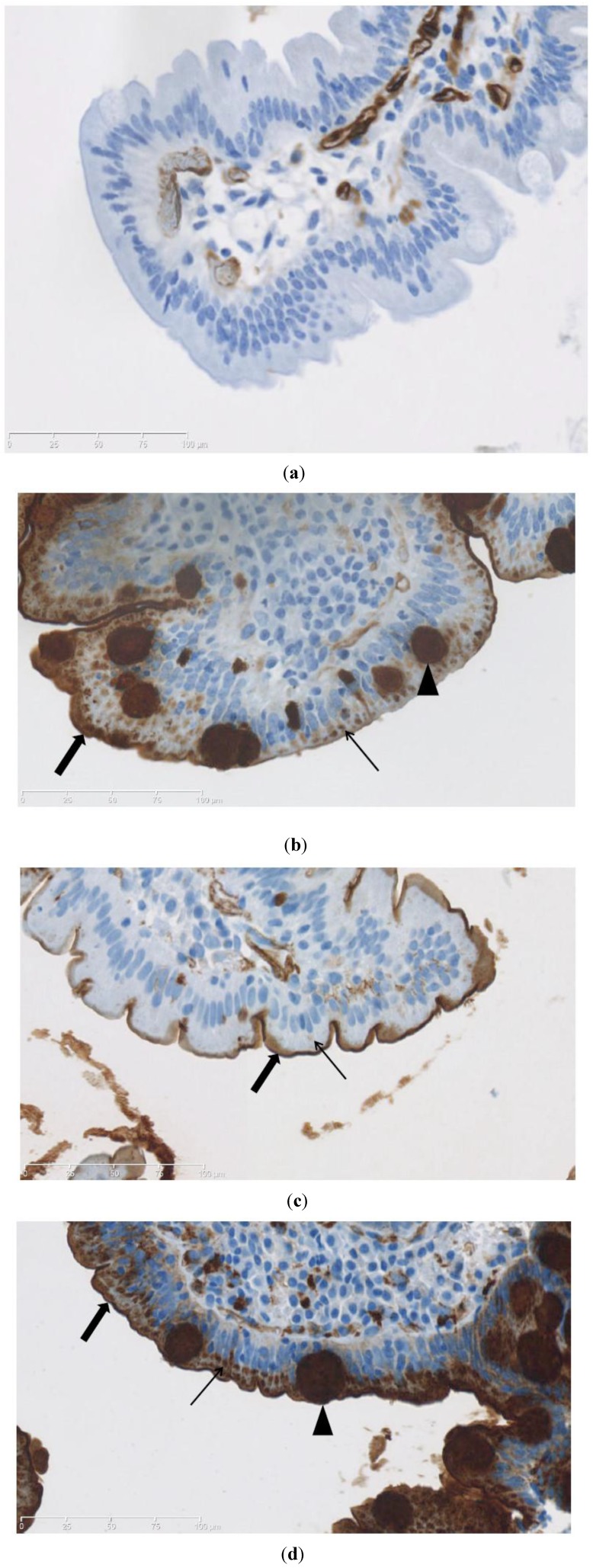
Ulex europaeus agglutinin (UEA) staining. (**a**) Patient C4 (non-CD), score 0; (**b**) Patient A1 (CD + T1D, untreated), score 3; (**c**) Patient A1 (CD + T1D, on GFD), score 2; (**d**) Patient B5 (CD), score 3. Thin arrows mark staining of cytoplasm of epithelial cells. Thick arrows mark staining of glycocalyx. Arrowheads mark staining in goblet cells.

UEA staining patterns are illustrated in Figure 2 with samples from a non-CD patient, a CD + T1D patient before and after treatment with GFD, and a CD patient. UEA staining was observed in all patients except one: C4 (Figure 2a). A score of 1 (cytoplasmic staining alone) was not given to any samples (Table 3). In general, the UEA staining appeared more intense in comparison to PNA staining. This was especially evident in goblet cells (Figure 2b,d).

**Table 3:** Ulex europaeus agglutinin lectin staining scores. Stained sections of biopsies from patients in Groups A (before and after remission), B, and C were scored as follows: 0 represents no staining of the epithelial cell layer, 1 represents staining in the cytoplasm of epithelial cells; 2 represents staining of cytoplasm and glycocalyx; and 3 represents staining of cytoplasm and glycocalyx, as well as goblet cells.

	Group	A CD + T1D Untreated	A CD + T1D GFD	B CD	C Non-CD
Score					
3		5	4	10	5
2		5	6	0	4
1		0	0	0	0
0		0	0	0	1
Median score		2.5	2	3	2.5

Scores from biopsy sections stained with PNA lectin are presented in Table 2. We performed three sets of statistical analysis to test if the scores reflected differences between the groups.

First, we compared the patients in Group A with both CD and T1D before (untreated) and after treatment with a GFD using the Wilcoxon signed-rank test for matched pairs. The median scores were 1.5 and 2 for the untreated and GFD-treated groups, respectively, with no statistically significant difference (*p* = 0.25).

Secondly, we compared the groups A untreated, B and C using the Kruskal–Wallis one-way ANOVA with Dunn’s post test and found no statistically significant differences between any of the groups.

Finally, we combined the results from the two groups with inflamed tissue (A untreated and B, median = 1) and compared with the combined results from the two groups without inflammation (A GFD and C, median = 1) in a Mann–Whitney test and found no statistically significant difference (*p* *=* 0.38). Likewise, there was no difference between the combined groups with CD (A untreated and B, median = 1) when compared with the non-CD group C (median = 1) (*p* = 0.88).

Scores from biopsy sections stained with UEA lectin are presented in Table 3. We performed statistical analyses analogous to the statistical tests described above for PNA lectin, and found no difference in the comparison between Group A untreated and GFD-treated (*p* = 1.00), no difference between any of the groups A untreated, B and C, and also no difference when the combined group with inflammation (median = 3) was compared with the combined group without inflammation (median = 2) (*p* = 0.09), or when the combined groups of CD patients (median = 3) were compared with the non-CD group C (median = 2.5) (*p* = 0.23).

Combining the results in Table 2 and Table 3 did not indicate any difference between the four groups in combined PNA/UEA staining with medians of 2, 2, 2.5 and 2, respectively.

## 4. Discussion

In our study, we found no difference in UEA and PNA lectin staining in biopsies from the same pediatric CD patients before and after remission. It should be noted that these children also had T1D, another autoimmune disease whereby the composition of commensals in the gastrointestinal tract could be of pathogenic importance [14]. Likewise, we found no difference when biopsies showing CD-associated inflammation were compared to biopsies with normal histology.

Earlier work has shown somewhat conflicting results regarding the reactivity of PNA in CD biopsies. PNA has been reported to not show any reactivity at all in patients or controls [15], to be upregulated in goblet cells of CD patients compared to controls [16], or downregulated in the glycocalyx of CD patients in a study where no reactivity in goblet cells was noted [8]. There is more agreement with regards to UEA reactivity, which was shown to be upregulated in goblet cells of CD patients in two of these studies [8,15]. Some methodological differences between these studies and our own could in part explain the discrepancies: whether it is children or adults being studied, the origin of the biopsy (jejunum as opposed to duodenum), and the specific structures included in the analysis (e.g., crypt goblet cells *versus* villus goblet cells).

It has been well described that the bacterial microbiota is important for the function of the mucosal immune system, and proper development of gut-associated lymphoid tissue is dependent on bacterial presence and influenced by the composition of the microbiota [17,18,19]. Colonization of germ-free mice with *Bacteroides* modulated the expression of numerousl genes involved in diverse intestinal functions including maintenance of intestinal permeability [20]. In rats, it was shown that the composition of the commensal microbiota can modulate intestinal permeability with certain bacterial strains increasing permeability, and other (probiotic) strains reducing permeability [21]. Colonization with such probiotic strains of bacteria could potentially reduce intestinal inflammation [22], and be beneficial in the context of celiac disease, whereas colonization with other strains could be detrimental.

Characterization of the bacterial population in the human gastrointestinal tract is currently intensely investigated [23], and it remains an important issue to define how the composition of the commensal microbiota is determined. It is clear that the exposure to diverse bacterial strains in different environments plays an important role, but a genetic predisposition to colonization with particular bacterial strains is also likely to influence the composition of the microbiota [24,25]. Such a genetic component could be the result of inherited differences in the expression pattern of unique carbohydrate structures in the glycocalyx/mucous layer. This could influence bacterial composition if bacteria can utilize carbohydrates for colonization, as was proposed for *Bacteroides* using glycans [26]. Recently, it was found that the genetically determined AB0 blood group system can modulate the composition of the human intestinal microbiota [27]. Furthermore, the secretor status encoded by the FUT2 gene, which defines the expression of the AB0 blood group antigens in the mucus, is associated with the composition of intestinal bifidobacteria [28]. If the AB0 antigens are present in the mucosal layer, they could facilitate bacterial colonization by acting as attachment sites or carbon sources. In a Finnish study, it was found that 14.7% of a healthy control population was homozygous for a nonsense mutation in the FUT2 gene leading to non-secretor status [29]. The non-secretor status was positively associated with CD susceptibility, and the frequency of non-secretors was increased to 18% in the CD population.

It appears likely that the composition of the intestinal microbiota is associated with the development of CD [30], either through a genetic predisposition, environmental influence, or infection with pathogens [31]. Several studies point to differences in the microbiota between CD patients with active disease, CD patients in remission, and normal controls [32,33,34]. Moreover, animal models have demonstrated how gluten-induced enteropathy can be modulated by the bacterial microbiota [35,36]. The presence of rod-shaped bacteria in intestinal mucosa was shown to be frequently associated with pediatric CD patients regardless of disease status (patients with active disease, compared to another group of patients in remission), but not with controls [8]. This was linked with differences in lectin staining of biopsies from the three groups in the study with UEA staining being more intense and widespread in CD patients (active CD or treated) compared to controls, and PNA staining being less widespread and intense in both groups of CD patients compared to controls. These findings could be explained by either a genetic predisposition to express certain carbohydrates in people who develop CD, or by the effect that the components of the microbiota, such as the rod-shaped bacteria associated with CD patients, could have on glycosylation.

Although not evident in our study, there is indication that inflammatory processes can alter the expression of carbohydrates in the gut. A characterization of the human MUC2 mucin in colon revealed a complex glycosylation pattern in healthy individuals [37]. This pattern was altered with a shift towards smaller and less complex glycans in patients with active ulcerative colitis [38]. Interestingly, this effect was reversed after remission of disease, arguing against a genetic reason for the shift.

With respect to CD in future studies, it would be relevant to investigate other differences in mucus glycosylation that are not detected by UEA or PNA staining. Also, potential differences in other modifications, such as sulfation, could be relevant in CD [39]. Our limited study supports the notion that glycosylation is independent of disease activity as previously shown by Forsberg and colleagues [8]. In contrast to that study, however, we could not demonstrate a difference in lectin staining between CD patients, either with or without T1D or combined, when compared to non-CD controls.

## 5. Conclusions

In our study, we did not identify any differences in the staining pattern of the lectins PNA or UEA in duodenal biopsies between samples obtained before and after remission in the same children with both CD and T1D. Also, we did not observe any differences in lectin staining between children with both CD and T1D, children with CD alone, or non-CD controls. In conclusion, we found no indication that the presence of unique carbohydrate structures bound by PNA or UEA is involved in the susceptibility to CD, or that the inflammation caused by the disease affects the glycosylation process.
